# *catena*-Poly[[(3,5-dicarb­oxy­pyrazine-2,6-dicarboxyl­ato-κ^3^ *O* ^2^,*N* ^1^,*O* ^6^)lithium(I)]-μ-aqua-[triaqua­lithium(I)]-μ-aqua]

**DOI:** 10.1107/S1600536810045903

**Published:** 2010-11-13

**Authors:** Wojciech Starosta, Janusz Leciejewicz

**Affiliations:** a Institute of Nuclear Chemistry and Technology, ul. Dorodna 16, 03-195 Warszawa, Poland

## Abstract

The title coordination polymer, [Li_2_(C_8_H_2_N_2_O_8_)(H_2_O)_5_]_*n*_ contains two symmetry-independent Li^+^ ions; one is coordin­ated by five water O atoms, the other by an *O*,*N*,*O*′-tridentate doubly deprotonated pyrazine-2,3,5,6-tetra­carboxyl­ate ligand and two water O atoms. Water mol­ecules bridge adjacent Li^+^ ions into ribbons propagating in [100]; an alternative analysis of the structure considers it to contain alternating [Li(C_8_H_2_N_2_O_8_)(H_2_O)_2_]^−^ anions and [Li(H_2_O)_3_]^+^ cations. In the polymeric model, both lithium ions show distorted trigonal–bipyramidal coordination geometries. Within the ligand, the carboxyl H atoms participate in short, almost symmetric O⋯H⋯O hydrogen bonds in which the non-coordinated carboxyl­ate O atoms are donors and acceptors. In the crystal, the ribbons inter­act *via* a network of O—H⋯O hydrogen bonds in which the coordinated water mol­ecules act as donors and ligand carboxyl­ate O atoms as acceptors.

## Related literature

For the crystal structures of 3*d* transition metal complexes with pyrazine-2,3,5,6-tetra­carboxyl­ate and water ligands, see: Alfonso *et al.* (2001 ▶); Graf *et al.* (1993 ▶); Marioni *et al.* (1986 ▶); Marioni *et al.* (1994 ▶). For the structure of a Ca(II) complex, see: Starosta & Leciejewicz (2008 ▶). For the structure of a Li complex with pyrazine-2,3-dicarboxyl­ate and water ligands, see: Tombul *et al.* (2008 ▶). For a review on metal organic frameworks (MOFs), see: MacGillivray (2010 ▶). 
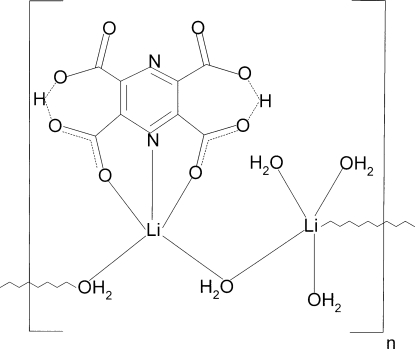


## Experimental

### 

#### Crystal data

[Li_2_(C_8_H_2_N_2_O_8_)(H_2_O)_5_]
*M* *_r_* = 358.08
Monoclinic, 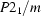

*a* = 6.8806 (14) Å
*b* = 11.767 (2) Å
*c* = 8.8082 (18) Å
β = 103.59 (3)°
*V* = 693.2 (2) Å^3^
*Z* = 2
Mo *K*α radiation
μ = 0.16 mm^−1^
*T* = 293 K
0.23 × 0.21 × 0.17 mm

#### Data collection

Kuma KM-4 four-circle diffractometer
Absorption correction: analytical (*CrysAlis RED*; Oxford Diffraction, 2008) ▶ *T*_min_ = 0.971, *T*_max_ = 0.975
2291 measured reflections
2136 independent reflections
1459 reflections with *I* > 2σ(*I*)
*R*_int_ = 0.012
3 standard reflections every 200 reflections  intensity decay: 0.5%

#### Refinement

*R*[*F* ^2^ > 2σ(*F* ^2^)] = 0.039
*wR*(*F* ^2^) = 0.127
*S* = 1.03
2136 reflections
148 parameters
H atoms treated by a mixture of independent and constrained refinement
Δρ_max_ = 0.42 e Å^−3^
Δρ_min_ = −0.32 e Å^−3^

### 

Data collection: *KM-4 Software* (Kuma, 1996 ▶); cell refinement: *KM-4 Software*; data reduction: *DATAPROC* (Kuma, 2001 ▶); program(s) used to solve structure: *SHELXS97* (Sheldrick, 2008 ▶); program(s) used to refine structure: *SHELXL97* (Sheldrick, 2008 ▶); molecular graphics: *SHELXTL* (Sheldrick, 2008 ▶); software used to prepare material for publication: *SHELXTL*.

## Supplementary Material

Crystal structure: contains datablocks I, global. DOI: 10.1107/S1600536810045903/hb5710sup1.cif

Structure factors: contains datablocks I. DOI: 10.1107/S1600536810045903/hb5710Isup2.hkl

Additional supplementary materials:  crystallographic information; 3D view; checkCIF report

## Figures and Tables

**Table 1:** Selected bond lengths (Å)

Li1—N1	2.119 (4)
Li1—O4	2.1194 (13)
Li1—O8	1.997 (4)
Li1—O7	2.075 (5)
Li1—O4^i^	2.1194 (13)
Li2—O6	1.9412 (12)
Li2—O7	2.385 (4)
Li2—O5	2.052 (4)
Li2—O6^i^	1.9412 (12)
Li2—O8^ii^	2.067 (4)

Symmetry codes: (i) 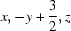; (ii) 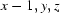.

**Table 2:** Hydrogen-bond geometry (Å, °)

*D*—H⋯*A*	*D*—H	H⋯*A*	*D*⋯*A*	*D*—H⋯*A*
O6—H61⋯O2^iii^	0.90 (2)	1.87 (2)	2.7597 (14)	165.4 (19)
O6—H62⋯O4^iv^	0.91 (3)	1.91 (3)	2.8129 (14)	173 (2)
O8—H81⋯O2^v^	0.86 (2)	1.92 (2)	2.7753 (13)	175.9 (19)
O7—H71⋯O3^iv^	0.862 (19)	2.037 (19)	2.8950 (12)	173.8 (17)
O5—H51⋯O1^iii^	0.903 (19)	2.010 (19)	2.9110 (13)	175.4 (17)
O1—H32⋯O3	1.19 (2)	1.21 (2)	2.3894 (15)	173 (2)

Symmetry codes: (iii) 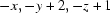; (iv) 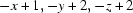; (v) 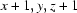.

**Fig. 1.**
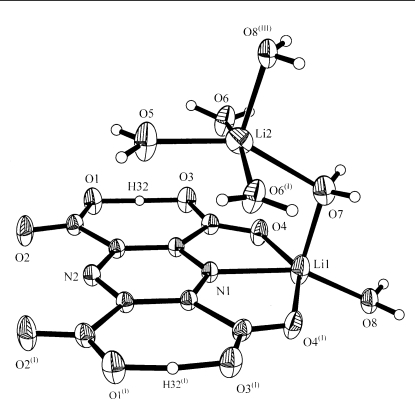
A structural unit of (1) with atom labelling scheme and 50% probability displacement ellipsoids. Symmetry code: (i) x, -y + 3/2, z; (ii) X+1, y, z; (iii) x - 1, y, z.

**Fig. 2.**
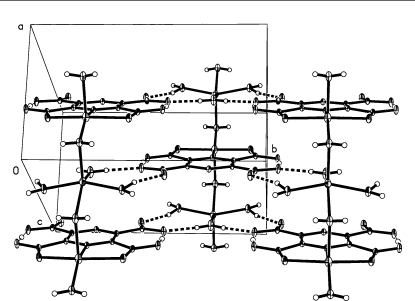
Packing diagram of the structure.

## supplementary crystallographic information

### Comment

Multidentate organic acids are widely used as ligands in a search for coordination polymers which can find applications in such fields as hydrogen or carbon dioxide storage, catalysis and ion-exchange resins (MacGillivray, 2010). Pyrazine-2,3,5,6-tetracarboxylate ligand is a promising building block in constructing metal-organic frameworks, since it exhibits ten chelating sites: two hetero-ring N atoms and four potentially bidentate carboxylate groups. A formation of a variety of catenated and layered polymeric structures has been reported in compounds of 3 transition metal ions with the title ligand (Marioni *et al.*, 1986; Graf *et al.*, 1993; Marioni *et al.*, 1994; Alfonso *et al.*, 2001). The Ca(II) complex shows a three-dimensional polymeric structure (Starosta & Leciejewicz, 2008). The asymmetric unit cell of a Li(I) complex with the title ligand contains two symmetry independent Li1 and Li2 ions positioned on a mirror plane with *y*=3/4. Pyrazine-ring N1 and N2 atoms and coordinated water O5, O7, O8 atoms are also positioned on this plane (Fig.1). The Li1 ion is chelated by the N1, O4, O4^i^ ligand bonding moiety with typical Li—N and Li—O bond distances of 2.119 (4)Å and 2.119 (1) Å, respectively and two bridging aqua O7 and O8 atoms (d_Li1—O7_ =2.075 (5)° and d_Li1—O8_=1.997 (4) Å). The O4 and O4^i^ atoms are at opposite apices of a distorted trigonal bipyramid; its equatorial plane is composed of coplanar Li1 ion, hetero-ring N1 atom and the bridging O7 and O8 atoms. Pyrazine-ring atoms are coplanar with r.m.s. of 0.0009 (1) Å; their plane makes an angle of 90° with the equatorial plane. Carboxylic groups C6/O1/O2 and C5/O1/O2 make angles with pyrazine ring of 1.45 (13)° and 4.69 (14)°, respectively. Bond distances and bond angles within the ligand molecule do not differ from those observed in other complexes with the title ligand. A proton situated between carboxylate O3 and O1 atoms, clearly visible on the Fourier map, forms a symmetrical intra-molecular hydrogen bond of 2.389 (2)Å and O3—H32—O1 angle of 173 (3)°. The (2-) charge of the ligand is compensated by the (1+) charge of the Li1 ion. The resulting anion has a charge of (1-). On the other hand, the Li2 ion as coordinated only by aqua O atoms, retains its charge of (1+). The ribbons (Fig. 2) are built of Li(C_8_H_2_N_2_O_8_)(H_2_O)_2_]^1-^ anions and [Li(H_2_O)_4_]^1+^cations bridged by aqua O7 and O8 atoms belonging to coordination spheres of both Li ions. The bridging pathway propagates with Li1—O7—Li2 angle of 111.19 (14)° and Li1—O8—Li2^iii^ angle of 103.81 (16)°. The coordination environment of the Li2 ion is composed of five aqua O atoms, two of them O7 and O8 are bridging it with the Li1 ions. The Li2 ion, O5, O7 and O8 atoms are coplanar and form an equatorial plane of a distorted trigonal bipyramid with O6 and O6^i^ atoms at opposite apices. Li—O bond distances within this coordination polyhedron are in the range from 1.941 (1)Å to 2.067 (4) Å, the Li2—O7 bridging bond distance amounts to 2.385 (4) Å. Trigonal bipyramidal coordination geometry of a Li(I) ion has been also observed in the structure of its complex with pyrazine-2,3-dicarboxylate and water ligands (Tombul *et al.* 2008). Coordinated water O atoms are donors in a network of hydrogen bonds to carboxylate O atoms in adjacent ribbons which act as acceptors.

### Experimental

50 ml of aqueous solution containing 1 mmol of pyrazine-2,3,5,6-tetracarboxylic acid was added to 50 ml of an aqueous solution containing 4 mmol of lithium hydroxide. The mixture was boiled under reflux for 3 h with conatant stirring, then left to crystallize at room temperature. After a couple of days crystals of (1) were found in the form of colourless blocks.  They were dissolved in water and recrystallized three times. The final crystals were washed in metanol and dried in the air.

### Refinement

Water H atoms were located in a difference map and refined isotropically.

### Crystal data

**Table:**

[Li_2_(C_8_H_2_N_2_O_8_)(H_2_O)_5_]	*F*(000) = 368
*M**_r_* = 358.08	*D*_x_ = 1.716 Mg m^−^^3^
Monoclinic, *P*2_1_/*m*	Mo *K*α radiation, λ = 0.71073 Å
*a* = 6.8806 (14) Å	Cell parameters from 25 reflections
*b* = 11.767 (2) Å	θ = 6–15°
*c* = 8.8082 (18) Å	µ = 0.16 mm^−^^1^
β = 103.59 (3)°	*T* = 293 K
*V* = 693.2 (2) Å^3^	Blocks, colourless
*Z* = 2	0.23 × 0.21 × 0.17 mm

### Data collection

**Table:**

Kuma KM-4 four-circle diffractometer	1459 reflections with *I* > 2σ(*I*)
Radiation source: fine-focus sealed tube	*R*_int_ = 0.012
graphite	θ_max_ = 30.1°, θ_min_ = 2.4°
profile data from ω/2θ scans	*h* = 0→9
Absorption correction: analytical (*CrysAlis RED*; Oxford Diffraction, 2008)	*k* = 0→16
*T*_min_ = 0.971, *T*_max_ = 0.975	*l* = −12→12
2291 measured reflections	3 standard reflections every 200 reflections
2136 independent reflections	intensity decay: 0.5%

### Refinement

**Table:**

Refinement on *F*^2^	Primary atom site location: structure-invariant direct methods
Least-squares matrix: full	Secondary atom site location: difference Fourier map
*R*[*F*^2^ > 2σ(*F*^2^)] = 0.039	Hydrogen site location: inferred from neighbouring sites
*wR*(*F*^2^) = 0.127	H atoms treated by a mixture of independent and constrained refinement
*S* = 1.03	*w* = 1/[σ^2^(*F*_o_^2^) + (0.0886*P*)^2^ + 0.040*P*] where *P* = (*F*_o_^2^ + 2*F*_c_^2^)/3
2136 reflections	(Δ/σ)_max_ < 0.001
148 parameters	Δρ_max_ = 0.42 e Å^−^^3^
0 restraints	Δρ_min_ = −0.32 e Å^−^^3^

### Special details

**Table:**

Geometry. All e.s.d.'s (except the e.s.d. in the dihedral angle between two l.s. planes) are estimated using the full covariance matrix. The cell e.s.d.'s are taken into account individually in the estimation of e.s.d.'s in distances, angles and torsion angles; correlations between e.s.d.'s in cell parameters are only used when they are defined by crystal symmetry. An approximate (isotropic) treatment of cell e.s.d.'s is used for estimating e.s.d.'s involving l.s. planes.
Refinement. Refinement of *F*^2^ against ALL reflections. The weighted *R*-factor *wR* and goodness of fit *S* are based on *F*^2^, conventional *R*-factors *R* are based on *F*, with *F* set to zero for negative *F*^2^. The threshold expression of *F*^2^ > σ(*F*^2^) is used only for calculating *R*-factors(gt) *etc*. and is not relevant to the choice of reflections for refinement. *R*-factors based on *F*^2^ are statistically about twice as large as those based on *F*, and *R*- factors based on ALL data will be even larger.

### Fractional atomic coordinates and isotropic or equivalent isotropic displacement parameters (Å^2^)

**Table:**

	*x*	*y*	*z*	*U*_iso_*/*U*_eq_	
N1	0.4556 (2)	0.7500	0.72520 (15)	0.0184 (3)	
N2	0.1994 (2)	0.7500	0.44032 (15)	0.0197 (3)	
O4	0.61711 (14)	0.92277 (7)	0.87982 (11)	0.0287 (2)	
O2	0.06363 (16)	0.93417 (8)	0.28320 (11)	0.0354 (3)	
O1	0.21919 (16)	1.05057 (7)	0.46536 (10)	0.0315 (3)	
O3	0.44557 (15)	1.04935 (7)	0.71677 (11)	0.0296 (2)	
C5	0.49353 (17)	0.94719 (9)	0.76029 (13)	0.0207 (2)	
C3	0.26124 (16)	0.84858 (8)	0.50943 (12)	0.0178 (2)	
C2	0.39451 (16)	0.84873 (8)	0.65765 (12)	0.0176 (2)	
C6	0.17301 (17)	0.95080 (10)	0.41030 (13)	0.0220 (2)	
O6	0.16203 (15)	0.91197 (8)	0.92116 (12)	0.0334 (3)	
O8	0.9298 (2)	0.7500	1.09008 (14)	0.0276 (3)	
O7	0.4555 (2)	0.7500	1.09036 (16)	0.0338 (3)	
O5	−0.0754 (3)	0.7500	0.72326 (19)	0.0438 (4)	
Li2	0.1189 (6)	0.7500	0.9392 (4)	0.0332 (7)	
Li1	0.6568 (5)	0.7500	0.9480 (4)	0.0347 (7)	
H61	0.071 (3)	0.9536 (18)	0.853 (2)	0.051 (6)*	
H62	0.230 (4)	0.962 (2)	0.992 (3)	0.079 (8)*	
H81	0.976 (3)	0.8072 (17)	1.148 (2)	0.054 (6)*	
H71	0.484 (3)	0.8066 (16)	1.154 (2)	0.044 (5)*	
H51	−0.125 (3)	0.8094 (16)	0.661 (2)	0.044 (5)*	
H32	0.336 (3)	1.055 (2)	0.588 (2)	0.061 (7)*	

### Atomic displacement parameters (Å^2^)

**Table:**

	*U*^11^	*U*^22^	*U*^33^	*U*^12^	*U*^13^	*U*^23^
N1	0.0231 (6)	0.0119 (5)	0.0173 (5)	0.000	−0.0007 (5)	0.000
N2	0.0242 (7)	0.0148 (6)	0.0177 (6)	0.000	0.0002 (5)	0.000
O4	0.0329 (5)	0.0198 (4)	0.0261 (4)	−0.0035 (3)	−0.0079 (4)	−0.0019 (3)
O2	0.0486 (6)	0.0218 (4)	0.0254 (4)	0.0038 (4)	−0.0124 (4)	0.0033 (3)
O1	0.0485 (6)	0.0135 (4)	0.0257 (4)	0.0029 (4)	−0.0046 (4)	0.0017 (3)
O3	0.0409 (5)	0.0126 (4)	0.0290 (4)	−0.0016 (3)	−0.0047 (4)	−0.0019 (3)
C5	0.0244 (5)	0.0145 (5)	0.0211 (5)	−0.0031 (4)	0.0008 (4)	−0.0018 (4)
C3	0.0220 (5)	0.0130 (5)	0.0166 (4)	0.0011 (4)	0.0011 (4)	0.0014 (4)
C2	0.0212 (5)	0.0130 (4)	0.0168 (4)	−0.0008 (4)	0.0010 (4)	−0.0005 (3)
C6	0.0282 (6)	0.0155 (5)	0.0201 (5)	0.0029 (4)	0.0011 (4)	0.0033 (4)
O6	0.0373 (5)	0.0181 (4)	0.0354 (5)	−0.0004 (4)	−0.0104 (4)	0.0004 (4)
O8	0.0340 (7)	0.0225 (6)	0.0212 (6)	0.000	−0.0037 (5)	0.000
O7	0.0486 (9)	0.0199 (6)	0.0288 (6)	0.000	0.0005 (6)	0.000
O5	0.0585 (10)	0.0214 (7)	0.0379 (8)	0.000	−0.0160 (7)	0.000
Li2	0.0433 (18)	0.0236 (15)	0.0341 (16)	0.000	0.0117 (14)	0.000
Li1	0.0393 (17)	0.0264 (15)	0.0295 (15)	0.000	−0.0099 (13)	0.000

### Geometric parameters (Å, °)

**Table:**

Li1—N1	2.119 (4)	N2—C3^i^	1.3324 (12)
Li1—O4	2.1194 (13)	O4—C5	1.2221 (15)
Li1—O8	1.997 (4)	O2—C6	1.2091 (15)
Li1—O7	2.075 (5)	O1—C6	1.2815 (15)
Li1—O4^i^	2.1194 (13)	O1—H32	1.19 (2)
Li2—O6	1.9412 (12)	O3—C5	1.2811 (14)
Li2—O7	2.385 (4)	O3—H32	1.21 (2)
Li2—O5	2.052 (4)	C5—C2	1.5279 (15)
Li2—O6^i^	1.9412 (12)	C3—C2	1.4081 (15)
Li2—O8^ii^	2.067 (4)	C3—C6	1.5260 (14)
Li2—Li1^ii^	3.199 (5)	O6—H61	0.90 (2)
Li1—Li2^iii^	3.199 (5)	O6—H62	0.91 (3)
O8—Li2^iii^	2.067 (4)	O8—H81	0.86 (2)
N1—C2^i^	1.3277 (12)	O7—H71	0.862 (19)
N1—C2	1.3277 (12)	O5—H51	0.903 (19)
N2—C3	1.3324 (12)		
			
C2^i^—N1—C2	122.10 (13)	O6—Li2—O5	90.18 (11)
C2^i^—N1—Li1	118.95 (6)	O6^i^—Li2—O5	90.18 (11)
C2—N1—Li1	118.95 (6)	O6—Li2—O8^ii^	100.61 (11)
C3—N2—C3^i^	121.05 (13)	O6^i^—Li2—O8^ii^	100.61 (11)
C5—O4—Li1	119.15 (11)	O5—Li2—O8^ii^	102.98 (18)
C6—O1—H32	116.2 (12)	O6—Li2—O7	84.17 (12)
C5—O3—H32	113.3 (11)	O6^i^—Li2—O7	84.17 (12)
O4—C5—O3	123.82 (10)	O5—Li2—O7	148.57 (19)
O4—C5—C2	117.04 (10)	O8^ii^—Li2—O7	108.45 (15)
O3—C5—C2	119.13 (9)	O6—Li2—Li1^ii^	100.02 (12)
N2—C3—C2	119.55 (9)	O6^i^—Li2—Li1^ii^	100.02 (12)
N2—C3—C6	112.55 (9)	O5—Li2—Li1^ii^	65.66 (13)
C2—C3—C6	127.91 (9)	O8^ii^—Li2—Li1^ii^	37.32 (10)
N1—C2—C3	118.88 (9)	O7—Li2—Li1^ii^	145.77 (15)
N1—C2—C5	110.38 (9)	O8—Li1—O7	106.53 (16)
C3—C2—C5	130.71 (9)	O8—Li1—O4^i^	102.54 (10)
O2—C6—O1	122.94 (11)	O7—Li1—O4^i^	96.41 (12)
O2—C6—C3	118.66 (10)	O8—Li1—O4	102.54 (10)
O1—C6—C3	118.40 (9)	O7—Li1—O4	96.40 (12)
Li2—O6—H61	119.4 (14)	O4^i^—Li1—O4	147.18 (17)
Li2—O6—H62	130.5 (17)	O8—Li1—N1	153.3 (2)
H61—O6—H62	106 (2)	O7—Li1—N1	100.14 (17)
Li1—O8—Li2^iii^	103.81 (16)	O4^i^—Li1—N1	74.03 (9)
Li1—O8—H81	122.3 (13)	O4—Li1—N1	74.03 (9)
Li2^iii^—O8—H81	100.2 (14)	O8—Li1—Li2^iii^	38.87 (10)
Li1—O7—Li2	111.17 (14)	O7—Li1—Li2^iii^	145.40 (16)
Li1—O7—H71	107.9 (13)	O4^i^—Li1—Li2^iii^	93.19 (12)
Li2—O7—H71	114.0 (13)	O4—Li1—Li2^iii^	93.19 (12)
Li2—O5—H51	129.1 (12)	N1—Li1—Li2^iii^	114.46 (17)
O6—Li2—O6^i^	158.1 (2)		

Symmetry codes: (i) *x*, −*y*+3/2, *z*; (ii) *x*−1, *y*, *z*; (iii) *x*+1, *y*, *z*.

### Hydrogen-bond geometry (Å, °)

**Table:**

*D*—H···*A*	*D*—H	H···*A*	*D*···*A*	*D*—H···*A*
O6—H61···O2^iv^	0.90 (2)	1.87 (2)	2.7597 (14)	165.4 (19)
O6—H62···O4^v^	0.91 (3)	1.91 (3)	2.8129 (14)	173 (2)
O8—H81···O2^vi^	0.86 (2)	1.92 (2)	2.7753 (13)	175.9 (19)
O7—H71···O3^v^	0.862 (19)	2.037 (19)	2.8950 (12)	173.8 (17)
O5—H51···O1^iv^	0.903 (19)	2.010 (19)	2.9110 (13)	175.4 (17)
O1—H32···O3	1.19 (2)	1.21 (2)	2.3894 (15)	173 (2)

Symmetry codes: (iv) −*x*, −*y*+2, −*z*+1; (v) −*x*+1, −*y*+2, −*z*+2; (vi) *x*+1, *y*, *z*+1.
